# Buffering Capacity and Effects of Sodium Hexametaphosphate Nanoparticles and Fluoride on the Inorganic Components of Cariogenic-Related Biofilms In Vitro

**DOI:** 10.3390/antibiotics11091173

**Published:** 2022-08-30

**Authors:** Caio Sampaio, Alberto Carlos Botazzo Delbem, Thayse Yumi Hosida, Ana Vitória Pereira Fernandes, Guilherme dos Santos Gomes Alves, José Antônio Santos Souza, Douglas Roberto Monteiro, Juliano Pelim Pessan

**Affiliations:** 1 Department of Preventive and Restorative Dentistry, School of Dentistry, Araçatuba, São Paulo State University (UNESP), Rua José Bonifácio, 1193, Araçatuba 16015-050, SP, Brazil; 2 Postgraduate Program in Health Sciences, University of Western São Paulo (UNOESTE), Presidente Prudente 19050-920, SP, Brazil

**Keywords:** phosphates, fluorides, biofilms, nanotechnology

## Abstract

Despite the remarkable effects of sodium hexametaphosphate nanoparticles (HMPnano) on dental enamel de-/re-mineralization processes, information on the effects of these nanoparticles on biofilms is scarce. This study assessed the effects of HMPnano, with or without fluoride (F), on the inorganic components and pH of *Streptococcus mutans* and *Candida albicans* dual-species biofilms. Solutions containing conventional/micro-sized HMP (HMPmicro) or HMPnano were prepared at 0.5% and 1%, with or without 1100 ppm F. A 1100 ppm F solution and pure artificial saliva were tested as positive and negative controls, respectively. The biofilms were treated three times and had their pH analyzed, and the concentrations of F, calcium, phosphorus, and HMP in the biofilm biomass and fluid were determined. In another set of experiments, after the last treatment, the biofilms were exposed to a 20% sucrose solution, and the biofilm pH and inorganic components were evaluated. The 1% HMPnano solution with F led to the highest biofilm pH, even after exposure to sucrose. The 1% HMPnano solution without F led to significantly higher phosphorus concentrations in comparison to all other groups. It can be concluded that 1% HMPnano and F influenced the biofilm pH, besides affecting most of the inorganic components of the dual-species biofilms.

## 1. Introduction

Dental caries is a multifactorial, dynamic disease, mediated by the dental biofilm and modulated by the diet [1], resulting from the acid production by the biofilm from carbohydrate metabolization [2]. Considering the dynamics involved in de- and re-mineralization processes, the pH of the biofilm fluid, as well as the concentrations of fluoride (F), phosphorus (P), and calcium (Ca) in the biofilm (solid and fluid phases) are known to exert a determinant role on the saturation of hydroxyapatite [3,4,5]. This has important clinical implications, as an inverse relationship between caries incidence and the inorganic composition of saliva and dental biofilm has been demonstrated [6].

The Gram-positive bacterium *Streptococcus mutans* plays a crucial role in dental caries onset and progression [7], especially due to its capacity to produce organic acids and to withstand stressful conditions such as low pH. It also has the ability to synthesize large amounts of extracellular polymers from sucrose metabolization, thereby promoting supportive conditions for the adhesion and aggregation of other acidogenic and aciduric microorganisms to the biofilm [8]. In addition to *S. mutans*, recent data have stated the role that the species *Candida albicans* exerts in caries dynamics, particularly due to the critical symbiotic activity between *S. mutans* and *C. albicans* [9,10], in which the production of polysaccharides by *C. albicans* seems to impact the development of *S. mutans* biofilms [9], which increases caries severity.

Despite the decline in caries incidence and prevalence worldwide [11], dental caries substantially affects several individuals and populations all over the world [2]. This has prompted the investigation of new strategies, including the supplementation of fluoridated products with different compounds, such as sodium hexametaphosphate (HMP), an inorganic cyclophosphate salt, which is known to increase the permeability of the microbial wall and to disperse the biofilm [12,13]. Recent data evaluating the activity of conventional/micro-sized HMP (HMPmicro) on dual-species biofilms of *S. mutans* and *C. albicans* demonstrated that a solution containing HMPmicro in combination with F substantially affected the inorganic components of the biofilms and promoted an enhanced buffering effect in comparison to a 1100 ppm F solution [14]. In addition, it was demonstrated that the incorporation of HMP nanoparticles (HMPnano) to fluoridated toothpastes led to superior protective effects against enamel demineralization, and reduced the production of extracellular polysaccharides by biofilms in situ, in comparison to fluoridated toothpastes containing HMPmicro [15]. Furthermore, this nanoparticle has been shown to promote substantial reductions in the cell viability, metabolism, and production of biomass, besides affecting the structure and the composition of the extracellular matrix of *S. mutans* and *C. albicans* dual-species biofilms [16].

Considering the promising effects of HMPmicro on the inorganic components of biofilms [14], in addition to the substantial protective activity of HMPnano on enamel [15] and on the viability and virulence of cariogenic-related biofilms [16], it would be interesting to evaluate the effects of HMPnano on the pH, and on the F, calcium (Ca), phosphorus (P), and HMP concentrations of biofilms. Thus, the aim of this study was to evaluate the effects of HMPnano, combined or not with F, on the pH and inorganic components of dual-species biofilms of *S. mutans* and *C. albicans*, prior to and after exposure to sucrose. The null hypothesis of this study was that the effect of HMP would not be influenced by its particle size, presence of F, or exposure to sucrose.

## 2. Results

### 2.1. Determination of the Biofilm pH

**Figure 1:**
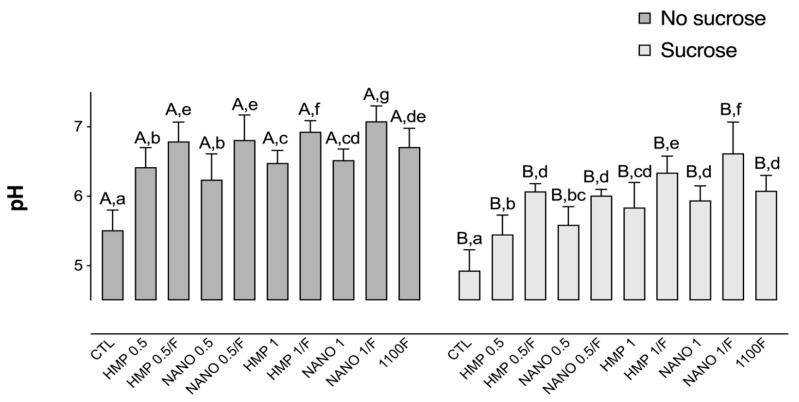
Mean pH values, prior to and after exposure to sucrose (cariogenic challenge). Distinct capital letters indicate statistical difference within each group regarding exposure to a 20% sucrose solution (in two levels—yes or no). Distinct lowercase letters indicate statistical difference among the experimental groups (all test solutions), within each condition of sucrose exposure). Data were subjected to two-way ANOVA, followed by Fisher’s LSD post hoc test for multiple comparisons (*p* < 0.05; n = 9). Bars denote standard deviations of the means. HMP: sodium hexametaphosphate; CTL: negative control; NANO: nano-sized HMP.

All test groups presented a significantly higher pH in comparison to the negative control, both prior to and after exposure to sucrose. In addition, biofilms treated with HMP or HMPnano combined with F presented a significantly higher pH in comparison to their counterparts without F, regardless of the exposure to sucrose. The NANO 1/F group showed the highest pH in comparison to all the other groups, followed by the HMP 1/F group (Figure 1). Exposure to sucrose promoted a significant decrease in the biofilm pH for all study groups, following a similar trend to that observed for biofilms not exposed to sucrose.

### 2.2. Analysis of the Inorganic Composition of the Biofilm Fluid

**Figure 2:**
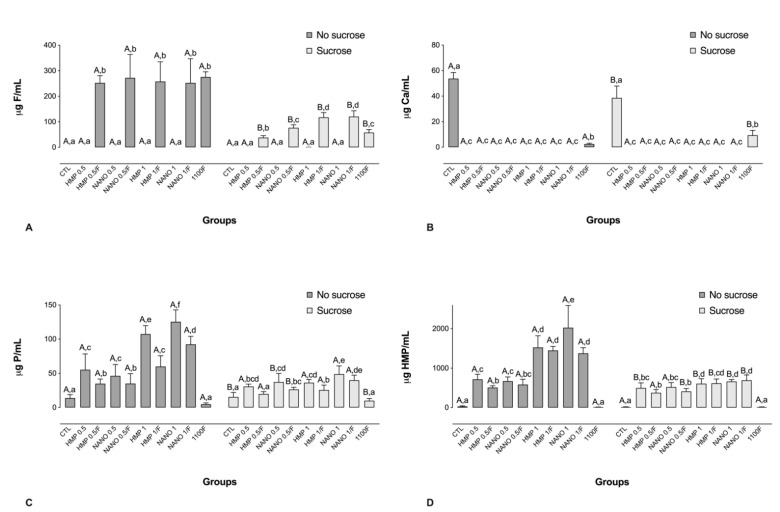
Mean values of F (**A**), Ca (**B**), P (**C**), and HMP (**D**) in the biofilm fluid, prior to and after exposure to sucrose (cariogenic challenge). Distinct capital letters indicate statistical difference within each group regarding exposure to a 20% sucrose solution (in two levels—yes or no). Distinct lowercase letters indicate statistical difference among the experimental groups (all test solutions), within each condition of sucrose exposure). Data were subjected to two-way ANOVA, followed by Fisher’s LSD post hoc test for multiple comparisons (*p* < 0.05; n = 9). Bars denote standard deviations of the means. Ca: calcium; F: fluoride; P: phosphorus; HMP: sodium hexametaphosphate; CTL: negative control; NANO: nano-sized HMP.

For the biofilms not exposed to sucrose, all groups treated with F-containing solutions had significantly higher F levels in comparison to the other groups (Figure 2A). After exposure to sucrose, biofilms treated with HMP 1/F or NANO 1/F presented the highest F concentrations, surpassing those attained by the use of 1100F (Figure 2A). Regarding Ca concentrations, exposure to phosphate-containing solutions led to significantly lower Ca levels in comparison to the negative and positive controls, which was not shown to be dependent on the particle size or exposure to sucrose (Figure 2B). In addition, the negative control group presented the highest Ca concentrations, followed by the positive control (Figure 2B).

Prior to the exposure to sucrose, biofilms treated with phosphate-containing solutions, regardless of particle size, led to significantly higher P concentrations in the biofilm fluid, in a dose-dependent manner. Furthermore, the association of HMP with F significantly reduced P levels in the biofilm fluid for all pairwise comparisons (Figure 2C). The highest P value was observed for NANO 1, which was significantly different from all the other groups except NANO 1/F (Figure 2C). A similar trend was observed for HMP in the biofilm fluid, both prior to and after exposure to sucrose (Figure 2D).

### 2.3. Analysis of the Inorganic Components from the Biofilm Biomass

**Figure 3:**
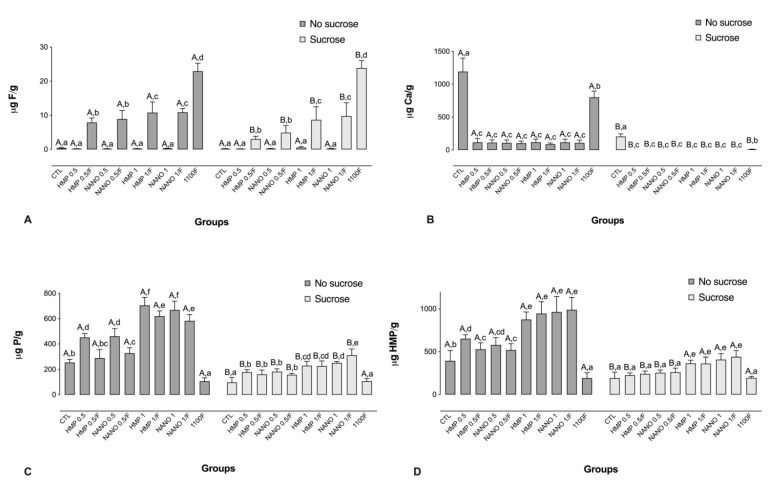
Mean values of F (**A**), Ca (**B**), P (**C**), and HMP (**D**) in the biofilm biomass, prior to and after exposure to sucrose (cariogenic challenge). Distinct capital letters indicate statistical difference within each group regarding exposure to a 20% sucrose solution (in two levels—yes or no). Distinct lowercase letters indicate statistical difference among the experimental groups (all test solutions), within each condition of sucrose exposure. Data were subjected to two-way ANOVA, followed by Fisher’s LSD post hoc test for multiple comparisons (*p* < 0.05; n = 9). Bars denote standard deviations of the means. Ca: calcium; F: fluoride; P: phosphorus; HMP: sodium hexametaphosphate; CTL: negative control; NANO: nano-sized HMP.

Treatment with 1100F led to the highest F levels, followed by treatments with HMPmicro or HMPnano at 1% combined with F, and HMPmicro or HMPnano at 0.5% combined with F. The exposure to sucrose promoted a significant decrease in F levels for all the F-containing groups (Figure 3A). Regarding Ca concentrations, a similar trend to that seen for the biofilm fluid was noted, in which the highest Ca levels were observed for the CTL, followed by 1100F. Moreover, prior to the exposure to sucrose, similar Ca levels were observed for all the HMP-containing groups, regardless of the presence of F or the particle size (Figure 3B).

A trend for a dose–response relationship was observed with regard to HMP concentrations of the test solutions and P levels in the biofilm biomass both prior to and after exposure to sucrose. In addition, without sucrose exposure, the addition of F to the HMP-containing solutions significantly reduced P levels in comparison to their counterparts without F (Figure 3C). A similar pattern was observed for HMP concentrations, albeit with a lesser impact of F (Figure 3D).

### 2.4. Determination of P Release from HMP

Mean (SD) P release from the solutions at 1, 7, and 30 days of the experiment was 10.5 (0.2), 10.9 (0.2), and 51.2 (7.3) µg P/mL for the HMP solutions, respectively, while the corresponding values for the NaH_2_PO_4_ solutions were 7968.4 (218.9), 7402.9 (364.9), and 7925.0 (156.2) µg P/mL, respectively.

## 3. Discussion

The present study was motivated by the remarkable effects of HMPmicro on the inorganic composition and pH of cariogenic biofilms in vitro [14], along with the higher reactivity of HMPnano over micrometric particles [16]. In comparison to HMPmicro, HMPnano had a higher buffering effect on dual-species biofilms of *S. mutans* and *C. albicans*, which was potentiated by F, and increased P levels in both the biofilm biomass and biofilm fluid, without reducing F levels in these compartments. On the other hand, HMP significantly reduced Ca levels in both compartments, and exposure to sucrose promoted an overall decrease in the values of all variables analyzed. Thus, the study’s null hypothesis was partially rejected.

Repetitive acid challenges resulting from carbohydrate metabolization by cariogenic biofilms lead to consecutive cycles of de- and re-mineralization at the biofilm–tooth interface, further leading to the development of caries lesions [2]. The present study showed that treatment with a solution containing only 1100 ppm F led to a significantly higher biofilm pH than that observed for the untreated biofilm (i.e., the negative control). This trend is in line with previous data that described the buffering capacity of F on *S. mutans* biofilms. In brief, HF formed at a low pH crosses the bacterial cell membrane and dissociates into H^+^ and F^−^ in the cytoplasm, which is more alkaline than the external environment [17]. The intracellular F^−^ then inhibits the activity of the glycolytic enzymes, leading to a reduction in the acid production from glycolysis. Furthermore, F^−^ present in the cytoplasm promotes a reduction in the cytoplasmatic pH by lowering the glycolytic activity, affecting both acid tolerance and production by *S. mutans* [18,19].

As for HMP, recent data demonstrated that the exposure to this polyphosphate substantially reduced biofilms’ acidogenicity in comparison to untreated biofilms [16] or in comparison to those exposed to F under highly cariogenic conditions [20]. In line with such data, the higher biofilm pH observed for the groups exposed to HMP-containing solutions is possibly related to the buffering ability of this phosphate due to its chelating property, according to which HMP in the biofilm fluid leads to the binding of HMP to H^+^, and of Na^+^ to OH^−^ [21]. The possible formation of HPO_4_^2−^ might also have played an important role, given its substantial buffering capacity [14,21]. In addition, these effects were further enhanced by the addition of F, given that combined treatments of HMP and F led to a significantly higher biofilm pH in comparison to their counterparts without F, for both concentrations and particle size of the phosphate. In fact, such an additive effect was even more notable when HMP was administered at the highest concentration, since treatments with HMP at 1% combined with F promoted a significantly higher pH in comparison to the positive control (i.e., biofilms treated with 1100 ppm F). Although the actual mechanism of the interaction of HMP and F is still unclear, recent data showed that co-administration of the two actives significantly increased the degree of saturation of HF° in the biofilm fluid in relation to hydroxyapatite, in comparison to the compounds alone [14].

The reduction in the particle size to the nanoscale was shown to enhance the buffering capacity of HMP at the highest concentration as well, even after exposure to sucrose. This superior effect of nanoparticles over micrometric ones is in line with a recent study using the same dual-species biofilm model, in which the biofilm metabolism, production of biomass, viability of *S. mutans*, and the protein and carbohydrate content from the extracellular matrix were significantly lower for solutions containing HMPnano compared with their micrometric counterparts [16]. This trend can be justified by the higher area/volume ratio and a higher percentage of atoms of nanoparticles, which make them more reactive than conventional ones [22,23].

Although HMP has a cyclic molecular structure, this study showed that HMP undergoes hydrolysis over time, which helps to explain the dose–response relationship between HMP concentrations in the test solutions and those in the biofilm. Interestingly, the concentrations of F and HMP in the biofilm biomass were significantly lower in groups treated with HMP/HMPnano co-administered with F, compared with the actives alone, suggesting that F and P from the treatments might have competed for the same binding sites. The effects above seem to be somehow linked to the superior buffering action of the actives when co-administered [14]. The reasons for such a trend, however, remain unclear, demanding further investigation of the topic.

Regarding Ca concentrations, it is noteworthy that biofilms exposed to HMP or HMPnano, regardless of the presence of F, led to negligible Ca concentrations in the biofilm fluid and biomass, which might be the result of the chelating capacity of HMP. It is well documented that phosphate presents a great affinity for cations [24,25,26], which results in the binding of HMP with these elements from the bacterial cell wall. This property provides HMP with an antimicrobial activity, which can affect parameters related to the biofilms such as the microbial viability and metabolism, as well as the expression of the components of the extracellular matrix [16,27]. The only source of Ca in the present protocol was the AS used as the growth medium, the reason for which the negative control group presented significantly higher concentrations of this ion compared with the other groups. Interestingly, treatments with a solution containing only F led to significantly higher Ca concentrations than the phosphate-containing treatments both in the biofilm fluid and biomass, but lower in comparison to the negative control. Such a trend may be explained by possible interactions between Ca and F, which was reflected in the biofilm pH as well.

Although the results presented in this work bring relevant information on the activity of HMPnano and F on biofilms, some drawbacks must be emphasized. Firstly, the data on the inorganic composition of the biofilms analyzed were not provided by biofilm–enamel substrate exchanges, which means that the ions evaluated were all from the culture medium (i.e., artificial saliva), so that direct extrapolations of these results to clinical conditions may not be completely appropriate. In addition, despite the two microbial strains (i.e., *S. mutans* and *C. albicans*) used in this study consisting of cariogenic-related microorganisms, dental caries is essentially a polymicrobial condition [28], so that future studies using multispecies biofilms could also bring important information on the activity of this nano-phosphate on more complex biofilms.

## 4. Materials and Methods

### 4.1. Biofilm Growth and Treatment of the Biofilms with the Experimental Solutions

*C. albicans* (ATCC 10231) colonies previously grown on Sabouraud dextrose agar (SDA; Difco, Le Pont de Claix, France) were suspended in 10 mL of Sabouraud dextrose broth (Difco), and aerobically incubated overnight at 120 rpm (37 °C). *S. mutans* (ATCC 25175) colonies grown on Brain Heart Infusion agar (BHI agar; Difco) were suspended in 10 mL of BHI broth (Difco) and statically incubated overnight (5% CO_2_ at 37 °C). After 24 h, *S. mutans* and *C. albicans* cells were recovered by centrifugation (at 5781× *g*, for 5 min), and washed twice with 10 mL of 0.85% NaCl (Sigma-Aldrich, St Louis, MO, USA). Thereafter, the concentrations of *C. albicans* cells were set at 10^7^ cells/mL using a Neubauer counting chamber, while *S. mutans* cells were adjusted in a plate reader at 640 nm (EON Spectrophotometer of EON, Biotek, Winooski, VT, USA) to 10^8^ cells/mL in artificial saliva [14,29]. The dual-species biofilms were grown in 6-well plates (Costar^®^ #3516, Corning Inc., Corning, NY, USA) in artificial saliva supplemented with sucrose (at 5% CO_2_, 37 °C), according to the following composition: 1 l demi-water, 4 g sucrose, 2 g yeast extract, 5 g bacteriological peptone, 1 g mucin type III, 0.35 g NaCl, 0.2 g CaCl_2_, and 0.2 g KCl; pH 6.8 [12,25]. All reagents for artificial saliva preparation were purchased from Sigma-Aldrich (St Louis, MO, USA). The growth medium (i.e., artificial saliva) was renewed once daily by removing half of the content of the wells (i.e., 2 mL) and adding the same volume of fresh artificial saliva [14].

The biofilms were grown for 72 h and treated for 1 min at 72, 78, and 96 h after the beginning of the biofilm formation (i.e., three treatments) [14]. The biofilms were treated with solutions prepared at concentrations determined based on previous studies evaluating the effects of HMPmicro and HMPnano on enamel de- and re-mineralization and on biofilms [16,27]. The experimental groups were: HMP at 0.5% (HMP 0.5), HMP at 1% (HMP 1), HMP at 0.5% in combination with 1100 ppm F (HMP 0.5/F), HMP at 1% in combination with 1100 ppm F (HMP 1/F), HMPnano at 0.5% (NANO 0.5), HMPnano at 1% (NANO 1), HMPnano at 0.5% in combination with 1100 ppm F (NANO 0.5/F), and HMPnano 1% in combination with 1100 ppm F (NANO 1/F). Moreover, artificial saliva was tested as a negative control (CTL) and a 1100 ppm F solution as a positive control (1100F). Sodium fluoride (NaF) was adopted as the F salt. HMP and NaF were purchased from Sigma-Aldrich, and all the experimental solutions were prepared in demi-water.

HMPnano was processed and characterized as described by Sampaio et al. [16]. In brief, HMP presented an amorphous phase, and no changes in the crystalline standard of the particles prior to (HMPmicro) or after (HMPnano) nano-synthesis, verified by X-ray diffraction analysis. In addition, HMPmicro presented an average size of 2.05 ± 0.69 µm, while the corresponding value for HMPnano was 0.38 ± 0.12 µm. In addition, both HMPmicro and HMPnano presented an overall trend of spherical particles [16].

### 4.2. Determination of the Biofilm pH

After the last treatment, the biofilms were gently washed with 1 mL artificial saliva (for 10 s), scraped from the wells with a cell scraper (Kasvi, São José dos Pinhais, Brazil), and transferred to microtubes with the aid of a pipette (MCT-200-C-Axygen). A pH electrode (PHR-146 Micro Combination pH Electrode—Fisher Scientific, Hampton, VA, USA), previously calibrated with standards of pH 7.0 and 4.0, was used for pH determination [14].

In another set of experiments, after the last treatment, artificial saliva was removed from the wells, and the biofilms were exposed to a 20% sucrose solution, for 3 min (as a cariogenic challenge) [14]. The sucrose solution was then removed and the biofilms were scraped and transferred to microtubes (within 1 min) for pH determination, exactly as described above [14].

### 4.3. Analysis of the Inorganic Composition of the Biofilm Fluid

The microtubes containing the biofilms scraped were centrifuged (at 15,267× *g*) at 4 °C, for 5 min, and the biofilm fluid was collected [14]. F concentration was analyzed by an ion-selective electrode (Orion 9409 BN) and reference electrode (Orion 900100), both coupled to a potentiometer (Orion–Thermo Scientific, Waltham, MA, USA). The electrodes were calibrated for F analyses using F standards of 0.09, 0.18, 0.36, 0.72, and 1.44 μg F/mL (for biofilms treated with F-free solutions) and 6.25, 12.5, 25, 50, and 100 μg F/mL (for biofilms treated with F-containing solutions). A total ionic strength adjustment buffer (TISAB II) was used at the same condition as for the samples, at a 1:1 ratio [14]. The Ca concentrations were determined by spectrophotometry on a plate reader (EON, BioTek, Winooski, VT, USA), at a 650 nm wavelength, as described by Vogel [30]. In brief, Arsenazo III was used as colorimetric agent. An aliquot of 5 μL in duplicate for both standards and samples, mixed with 50 μL Arsenazo III and 50 μL demi-water, was shaken for 60 s in a plate reader prior to obtaining the resulting absorbance. As for P analyses, this was determined according to Fiske & Subbarow [31], while for determining the HMP concentration, this was performed after 1 h of water bath heating at 100 °C [14]. For the samples exposed to sucrose, HMP concentration was determined after 6 h of water bath heating at 60 °C [14]. The values obtained in millivolt (for F analysis) or absorbance (for Ca, P, and HMP analysis) were converted into μg using the Microsoft Excel software (Version 2010; Microsoft Corp., Redmond, WA, USA), allowing the determination of the analytical parameters (linearity, slope, and coefficient of variation).

### 4.4. Analysis of the Inorganic Composition of the Biofilm Biomass

For the evaluation of F, Ca, and P concentrations in the biofilm biomass, a 10.0 mg wet weight plaque was weighed on a scale (Shimadzu AUY 220, Kyoto, Japan) in microtubes, and 0.5 mol/mL HCl was added to the microtubes and further homogenized [32]. The resulting mixture was kept for 3 h at room temperature under constant shaking (120 rpm), and centrifuged (at 11,000× *g*), for 1 min. Thereafter, 400 μL of the liquid was removed and 400 μL 0.5 mol/L NaOH was added to the liquid to neutralize it [33].

The F concentration of the biofilm biomass was analyzed as described above for the biofilm fluid, using F standards containing 0.09, 0.18, 0.36, 0.72, and 1.44 μg F/mL (for the biofilms treated with F-free solutions), and 0.8, 1.6, 3.2, 6.4, and 12.8 μg F/mL (for the biofilms treated with F-containing solutions). The concentrations of Ca, P, and HMP were determined exactly as described above for the biofilm fluid.

### 4.5. Determination of P Release from HMP

In order to assess the rate of P release from HMP, aqueous solutions containing 1% HMP (test) and 3% NaH_2_PO_4_ were prepared and kept at 37 °C. The rate of release of free P from the solutions was assessed at 1, 7, and 30 days after preparation, according to Fiske & Subbarow [31], as described above.

### 4.6. Statistical Analysis

All experiments were run in biological triplicate, on three different occasions (n = 9). Data were subjected to a two-way ANOVA, considering exposure to sucrose (in two levels) and study groups (in ten levels) as variation factors. The Fisher’s LSD test was used as a post hoc test for ANOVA to indicate differences between groups (sucrose exposure within each test solution) and among groups (test solutions within each condition of sucrose exposure). Statistical analysis was conducted using SigmaPlot 12.0 software (San Jose, CA, USA), adopting *p* < 0.05.

## 5. Conclusions

It can be concluded that (1) HMP at 1% combined with fluoride influenced the pH of the biofilms analyzed, besides affecting most of the inorganic components of the dual-species biofilms, and (2) the reduction in the size of the particles of HMP to the nanoscale led to an enhancement in its buffering capacity. The data presented in this study contribute to the knowledge of how HMPnano and fluoride act on cariogenic-related biofilms and, along with the existing body of evidence, attest the potential use of this association on caries prevention and control.

## Data Availability

The data presented in this study are available on request from the corresponding author.
